# Differential proteomics of tobacco seedling roots at high and low potassium concentrations

**DOI:** 10.1038/s41598-021-88689-4

**Published:** 2021-04-28

**Authors:** Lin-jian Dai, Yu-kun Liu, Chong-wen Zhu, Jun Zhong

**Affiliations:** 1 College of agriculture of Hunan Agriculture University, Changsha, 410128 Hunan People’s Republic of China; 2 Tobacco Monopoly Bureau of Shimen County of Changde City, Changde, 415300 Hunan People’s Republic of China

**Keywords:** Biological techniques, Plant sciences

## Abstract

The effects of high potassium and normal potassium treatments on protein expression in roots of flue-cured tobacco plant HKDN-5 at the seedling stage were analyzed by an unlabeled protein quantification technique. The results showed that 555 proteins were differentially expressed (245 proteins were down-regulated and 310 proteins were up-regulated) in high potassium treatment compared with normal potassium treatment. Differentially expressed proteins were involved in 96 metabolic pathways (42 metabolic pathways, 21 synthetic pathways as well as catabolic pathways, including fatty acid metabolism, phenylpropane biosynthesis, ketone body synthesis and degradation, and butyric acid metabolism. Root processing of high potassium concentrations leads to increases in the synthesis of peroxidase, superoxide dismutase and acyl-coenzyme-A synthetase. Additional proteomic differences observed in tobacco roots grown in high potassium include proteins involved with genetic information processing as well as environmental sensing. Examples include RNA helicase, ABC transporters and large subunit GTPases. These up-regulated differentially expressed proteins function mainly in protein translation, ribosome structure and protein synthesis. This indicates that under high potassium treatment, root protein synthetic processes are accelerated and substance metabolism pathways are enhanced; thus, providing the material and energetic basis for root growth.

## Introduction

Potassium is the most important and abundant cation in plant cells. Potassium plays an important role in many physiological processes of plants, such as enzyme activation^1,2^, stomatal opening and closing^3^, membrane transport, charge balance, osmotic adjustment^4,5^. At the same time, as an important nutrient element, potassium also participates in important life activities, including photosynthesis, transportation and distribution of assimilates, which have an important impact on the growth and development of plants. Tobacco is a typical potassium loving crop and potassium plays an important role in improving the maturity, combustibility, smoking quality, and safety of cigarette products (for example, by reducing the production of tar and other harmful compounds)^6^. The root system is an important organ for flue-cured tobacco to absorb water and nutrients from soil and to synthesize hormones. Root system development directly affects the subsequent appearance and growth of tobacco plants, and affects the yield and quality of tobacco^7^. Therefore, it is of great significance to study the changes in root proteins under different potassium levels for practical cultivation applications in flue-cured tobacco.

In recent years, there has been more and more research on proteomics in tobacco, which mainly focused on the study of differential proteins under stress. For example, the protein levels in tobacco were studied after infection with the wildfire pathogen to analyze the wildfire disease resistance mechanism. Differences were detected in ten proteins; six had decreased expression, four had increased expression, and the ways in which the functions of the differentially expressed proteins are involved in the disease-resistance mechanism are discussed by Cui^9^. Another study used Cuibi No.1 tobacco as the test material; root and leaf proteins under low and high nitrogen supply levels were systematically analyzed by comparative proteomics^8^, to understand the changes in protein expression and abundance in roots and leaves under low nitrogen stress. This study further analyzed the response mechanism of multiple metabolic pathways in tobacco to a low nitrogen environment on the basis of protein expression. However, there are few reports that analyze the differences in protein abundance in roots of tobacco seedlings grown at high or low potassium concentrations.

Label free quantitative proteomics technologies can generally be divided into two methods: one is a signal intensity method based on peak height, peak area and peak capacity of the spectrum; the other is a spectrum counting method based on the number of secondary spectrums of matching peptides. In theory, label free proteomics can be used for protein quantitative analysis of any sample, and it can be used to compare quantitative data of the same sample from different sources, with high data portability and wide adaptability.

In this study, hydroponic experiments and label free protein quantitative technology were used to explore the expression of proteins in roots of flue-cured tobacco seedlings under high potassium and normal potassium conditions. This will provide a theoretical basis for the study of the location and function of the proteins and related enzymes differentially expressed under these two conditions, and their possible differential effects on flue-cured tobacco plants.

## Materials and methods

### Materials

HKDN-5is a new flue-cured tobacco line with stable characters, which was bred by one of the authors of the present research (Dai lin-jian. of Hunan Agricultural University) in 2003 through distant hybridization and selfing homozygosity for more than 10 years. It’s outstanding characteristic is a high potassium content compared with other varieties^10^.

### Methods

#### Material planting

The experimental materials were hydroponically grown in the greenhouse in Hunan Agricultural University Yunyuan science and education base at 2018, here the tobacco variety was HKDN-5. The treatments were designated high potassium (K^+^ concentration of 720 mg·L^−1^) and normal potassium (K^+^ concentration of 240 mg·L^−1^, control). The concentration of potassium in Hoagland's nutrient solution was increased by potassium nitrate, and the content of nitrogen was decreased by ammonium nitrate; during hydroponics, the ambient temperature was controlled at 25 ± 2 °C.

#### Sample collection, protein preparation and concentration determination

When the seedlings of HKDN-5 grew to 5–6 leaves (before transplanting), 4–5 plants with normal growth were selected and their roots were combined to make a mixed sample. Each treatment was replicated 3 times. Samples were frozen in liquid nitrogen and kept frozen until use.

Proteins from tobacco leaves were extracted with a phenol method^11^, and protein content was determined by the Bradford method^12^.

#### Proteolysis

100 μgs of each sample was transferred to a test tube and adjusted to a constant volume of 100 μL with 8 M urea. 11 μLs of 1 M DTT were added and incubated at 37 °C for 1 h, Next, the samples were transferred to 10 K ultrafiltration tubes and centrifuged at 14,000 *g* for 10 min. 120 μL of 55 mM iodoacetamide were added after centrifugation, and then the samples were incubated at room temperature for 20 min in the dark. In the same ultrafiltration tube, the samples were centrifuged for three times with 100 mM TEAB to replace the urea system. After this operation, trypsin was added at aprotein: enzyme ratio of 50:1. Enzymolysis was conducted overnight, after which the samples were freeze-dried^13^.

#### Nano-HPLC–MS/MS analysis

The lyophilized peptides were redissolved in 30 μL formic acid solution and separated by nano-LC, and analyzed by on-line electrospray ionization tandem mass spectrometry. The experiment was carried out on the Nano ACQUITY UPLC system, and the system was connected with a Q-Exactive mass spectrometer equipped with an on-line nano electrospray ion source. A 10 μL peptide sample was loaded into a capture column at a flow rate of 10 μL/min and then separated on a linear gradient: 3% a to 32% a (A: 0.1% formic acid ACN solution) within 120 min. The column was equilibrated under initial conditions for 10 min. The column flow rate was controlled at 300 nL/min, and the electrospray voltage was 2 kV^14^.

#### Data analysis

The peptides were filtered with a 1% FDR and 1 unique peptide. According to the ANOVA algorithm, proteins with significance greater than 13 (p-value less than 0.05) were selected as differentially expressed proteins.

Go was used to analyze the molecular functions, cellular locations and biological processes of proteins; cog & KOG annotation analysis was used to predict and classify protein functions and metabolic pathways. Metabolic pathways with P value < 0.05 were used to analyze the biological process of proteins^15^.

## Results and analysis

### Flue-cured tobacco seedling root phenotypic differences

**Figure 1:**
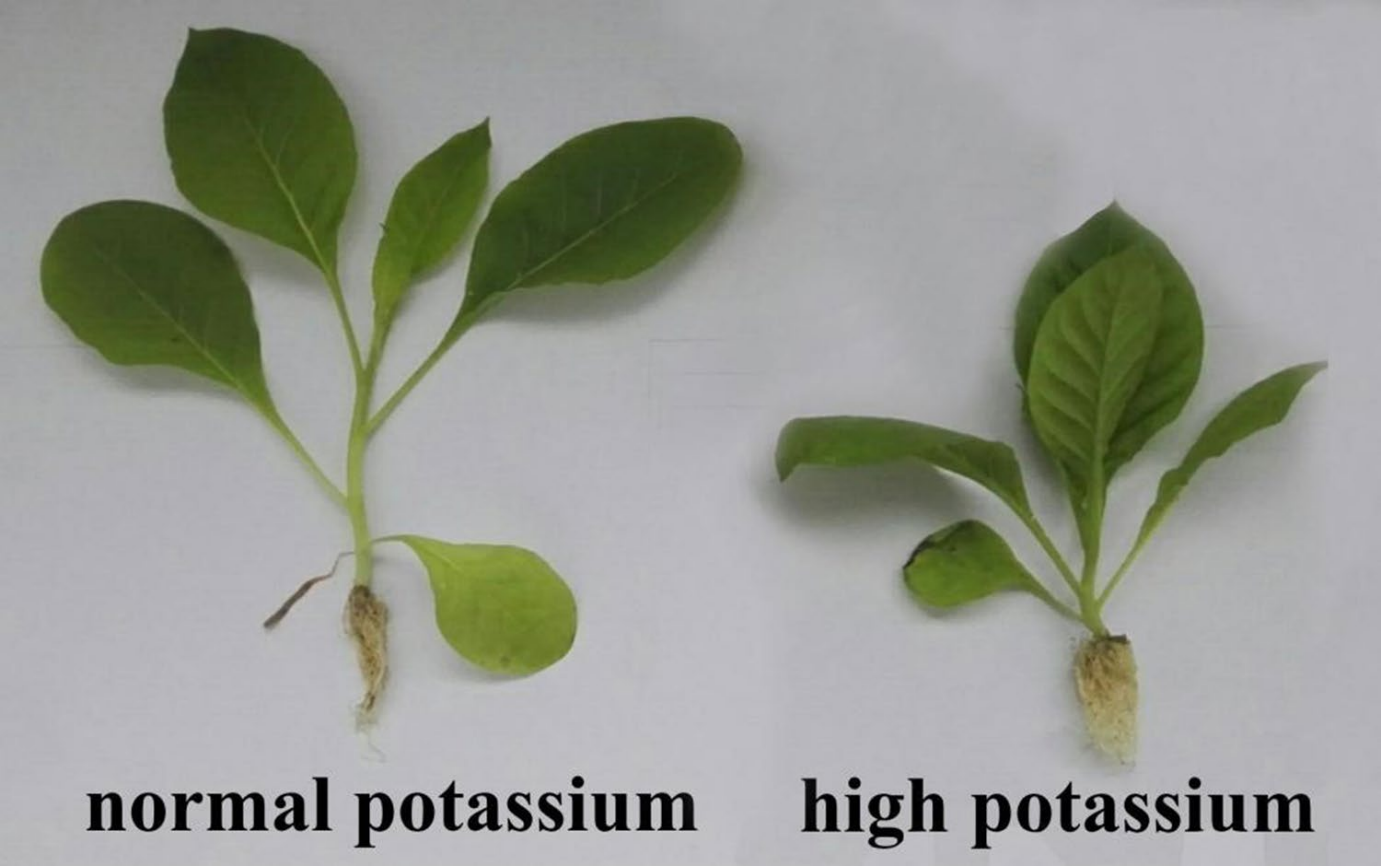
Phenotypic differences of tobacco seedlings grown at two potassium concentrations.

The root length of tobacco seedlings treated with a high concentration of potassium is shorter than the root length of control tobacco seedlings. However, at high potassium, the number of fibrous roots are more than the control treatment and the diameter of the taproots are greater than the control treatment. Meanwhile, the dry weights and fresh weights of the roots of the high potassium grown seedlings is greater than the control treatments (Table 1 and Fig. 1). Therefore, growth in a high concentration of potassium leads to a more developed root system of flue-cured tobacco seedlings than growth in a low concentration of potassium and provides a better material basis for the growth of tobacco plants.

**Table 1:** Phenotype difference on root of flue-cured tobacco at seedling under two potassium treatment.

Treatment	Fibrous root number	Longest root length/cm	Diameter of main root/cm	Root wet weight/(g·plant^-1^)	Root dry weight/(g·plant^-1^)
Normal potassium	48.5	6.34	0.088	0.883	0.042
High potassium	66.5	6.21	0.113	1.429	0.067

### SDS-PAGE electrophoresis of roots at seedling stage

**Figure 2:**
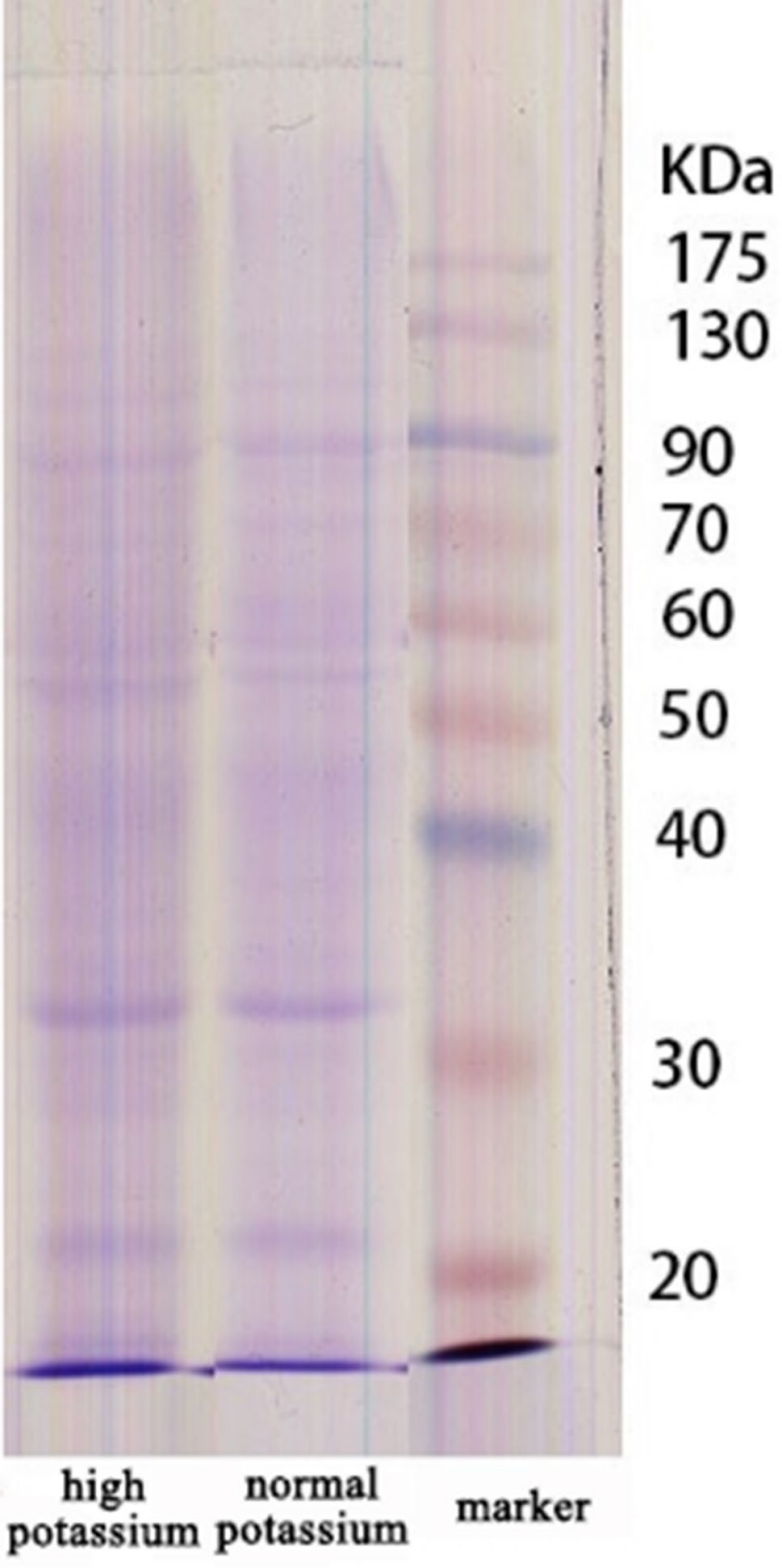
SDS-PAGE electrophorogram of root protein of flue-cured tobacco at seedling under two potassium treatment.

The distribution of total proteins in root cells of high potassium and normal potassium treated seedlings was uniform, with similar protein bands and no high abundance proteins (Fig. 2), which met the requirements of GC–MS detection for protein abundance and was convenient for differential proteomics analysis.

### Differential protein identification and statistics

Compared with the control treatment, 555 differentially expressed proteins in roots were found under high potassium treatment (differential expression greater than 1.5, P < 0.05). Among these 245 were down regulated and 310 were up regulated. Up regulated and down regulated differentially expressed proteins were mainly concentrated in the difference multiple range of 1.5–2, accounting for 61.2% and 54.2% of the total down regulated and up regulated differentially expressed proteins (Table 2).

**Table 2:** The number of differentially expressed proteins in tobacco roots after different multiplus (DT) of growing seedlings in high potassium medium, compared with growing seedlings in low potassium control medium.

	1.5 ≤ DT < 2	2 ≤ DT < 4	4 ≤ DT < 8	DT ≥ 8	Total
Up regulated protein	150	79	12	4	245
Down regulated protein	168	110	23	9	310
Total	318	189	35	13	555

### Bioinformatics analysis

#### GO annotation of differential proteins

Compared with the control treatment, 73% of the differential proteins were located in cell components (39%), membranous organelles (20%) and organelles (14%). In terms of molecular function, most of the differentially expressed proteins have the functions of organic ring compound binding (19%), heterocycle compound binding (19%), ion binding (18%), hydrolase activity (10%) and small molecule binding (10%). This accounts for 76% of the total differentially expressed proteins. In terms of biological processes, 71% of differential proteins are involved in cell metabolism (15%), organic metabolism (15%), basic metabolism (13%), nitrogen component metabolism (10%) and monomer metabolism (10%) (Table 3).

**Table 3:** GO annotation and percentage of differential expressed proteins in high potassium treatment.

Cellular component	%	Molecular function	%	Biological process	%
Cell part	39	Organic cyclic compound binding	19	Cellular metabolic process	15
Membrane-bounded organelle	20	Heterocyclic compound binding	19	Organic substance metabolic process	15
Organelle part	14	Ion binding	18	Primary metabolic process	13
Non-membrane-bounded organelle	9	Hydrolase activity	10	Nitrogen compound metabolic process	10
Ribonucleoprotein complex	8	Small molecule binding	10	Single-organism metabolic process	10
Cell–cell junction	4	Carbohydrate derivative binding	7	Single-organism cellular process	9
Whole membrane	2	Structural constituent of ribosome	4	Biosynthetic process	8
Virion part	1	Lyase activity	4	Catabolic process	5
DNA packaging complex	1	Cofactor binding	3	Cellular component organization	4
Transporter complex	1	Isomerase activity	2	Response to stress	3
Extracellular region part	1	Ligase activity	2	Cellular component biogenesis	3
		Sulfur compound binding	1	Response to chemical	2
		Macromolecular complex binding	1	Cell wall organization	2
				Cellular localization	1

#### KOG annotation of differential proteins

**Figure 3:**
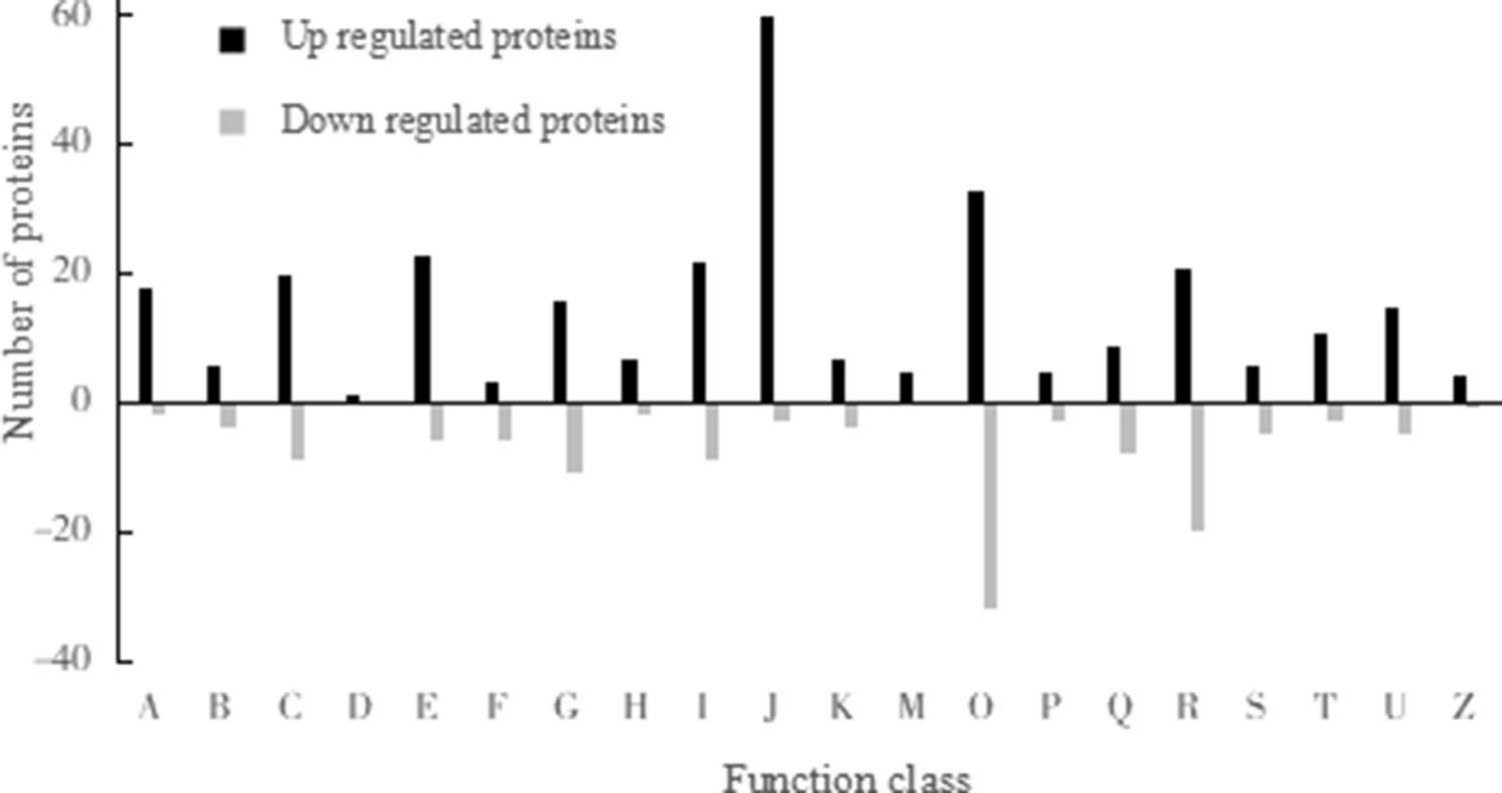
KOG annotation of differentially abundant proteins in high potassium treatment.

Compared with the control treatment, the proteins differentially expressed under high potassium treatment have 20 different functions:RNA processing and modification (A), chromosome structure and dynamics (B), energy generation and conversion (C), cell cycle regulation, cell division and chromosome division (D), amino acid transport and metabolism (E), nucleotide transport and metabolism (F), carbohydrate transport and metabolism (G), coenzyme transport and metabolism (H), lipid transport and metabolism (I), translation, ribosome structure and biological origin (J), transcription process (K), biological origin of cell wall, cell membrane and envelope (M), post-translational modification, protein conversion, chaperone (O), inorganic ionophore transport and metabolism (P), secondary metabolite biosynthesis transport and catabolism (Q),general function (R), unknown function (S), signaling mechanism (T), intracellular transport, secretion and vesicular transport (U), cytoskeleton and other related proteins (Z) (Fig. 3).

The most up-regulated functional class was (J), the least was (D), and the most down-regulated functional class was (O). Compared with the control treatment, the differentially expressed proteins in the high potassium treatment mainly belonged to translation, ribosome structure and biological origin (J),and post translational modification, protein conversion, and chaperone protein (O) functional classes.

Of the most differentially abundant proteins in the high potassium treatment (≥ fourfold difference) there were15 proteins (13 up-regulated and 2 down-Regulated) that are involved in metabolism related processes such as C, E, I, P, Q and H, 6 proteins (5 up-regulated and 1 down-regulated) that are involved in genetic information processing related processes such as A, B and J, 4 proteins (1 up-regulated and 3 down-regulated) that are involved in cell and environmental information processing related processes such as M, O and T, and 9 proteins (8 up-regulated and 1 down-regulated) that are involved in R and S Processes (Table 4).

**Table 4:** Identification of differentially expressed proteins in high potassium treatment.

Function	Gene.Name	Unique peptide	Protein Name	Fold.Change	KOG.Fun
Metabolism related	LOC107808039	4	Cytochrome b	4.72	C
	LOC107822887	11	Glutamate synthase	4	E
	LOC107804365	3	Isocitrate dehydrogenase	18.75	E
	LOC107797411	5	delta-1-pyrroline-5-carboxylate synthase	6.46	E
	LOC107798726	5	Peroxisomal	4.74	I
	LOC107832497	4	Phospholipase	− 4.72	I
	LOC107783543	3	Acyl-CoA synthetase	23.41	I
	LOC107768675	2	Copper transport	4.66	P
	LOC107800004	2	Superoxide dismutase	4.56	P
	LOC107827847	5	Alcohol dehydrogenas	8.21	Q
	CYP74D3	4	9-divinyl ether synthase 9	8.47	Q
	LOC107797980	2	UDP-glycosyltransferase UDP	6.7	GC
	LOC107822067	4	Serine carboxypeptidase	− 4.72	OE
Genetic information processing related	LOC107784716	2	RRM motif-containing protein	4.68	A
	LOC107793153	2	ATP-dependent RNA helicase	4.28	A
	LOC107816023	3	ATP-dependent RNA helicase	6.93	A
	LOC107807147	3	Histone	5.32	B
	LOC107807454	2	apoptotic chromatin condensation inducer in the nucleus	− 13.22	B
Cell process and environmental information processing related	LOC107830010	2	Sucrose synthase	4.19	M
	LOC107824172	2	T-complex 1 subunit T	− 4.29	O
	LOC107826414	2	Apoptotic ATPase	− 4.69	T
	LOC107822067	4	Serine carboxypeptidase	− 4.72	OE
Others	LOC107822201	5	Chitinase	13.45	R
	LOC107827351	6	Serine–glyoxylate aminotransferase	− 4.37	R
	LOC107764011	2	High-glucose-regulated protein	4.19	R
	LOC107790074	2	ABC transporter	7.2	R
	LOC107795389	3	Clustered mitochondria	5.41	R
	LOC107832395	2	N-alpha-acetyltransferase	4.23	R
	LOC107795579	2	Large subunit GTPase	4.78	R
	LOC107819873	2	Conserved protein	9.79	S

Fold changes are positive numbers indicating up regulated proteins, negative numbers indicate down-regulated expression.

#### KEGG pathway analysis of differentially abundant proteins

The results showed that compared with the control, the proteins that are differentially expressed under high potassium treatment participated in 96 pathways (42 metabolic pathways, 21 synthetic pathways and other catabolic pathways), including fatty acid metabolism, phenylpropane biosynthesis, ketone synthesis and degradation, butyric acid metabolism, etc. The root proteins differentially expreessed under high potassium treatment mainly participated in the process of material metabolism, which provided the basis for the better growth and development of the roots.

## Discussion

### Differentially abundant proteins related to metabolism

Analysis of the high potassium treatment compared with the normal treatment suggested that the main differentially abundant proteins related to metabolism are serine carboxypeptidase, phospholipase, alcohol dehydrogenase, peroxidase, superoxide dismutase, acyl coenzyme A synthetase, etc.

Serine carboxypeptidase is a kind of protease belonging to the α/β hydrolase family, which plays a significant role in the biosynthesis of secondary metabolites, the catalysis of acyl transfers and the degradation of seed germination related proteins. This enzyme gene is a kind of stress resistance gene of plants, which plays a significant role in plant growth and development and stress resistance^16,17^. Phospholipase plays roles in various biological and abiotic stress signal transduction pathways. It can hydrolyze phospholipid and participate in other lipid metabolism pathways as main raw materials after hydrolysis, or directly participate in stress response signal transduction as signal molecules^18,19^. Our results that these two enzymes are down regulated in the high potassium environment may be due to the fact that these enzymes are more conducive to the distribution and utilization of substances or are not needed in the non-adverse high potassium environment.

Alcohol dehydrogenase is the leading enzyme of ethanol fermentation, which plays a vital role in the anaerobic respiration of plants. It is an important hydrolase in the abnormal respiratory chain of plants, and it is also closely related to the stress resistance physiology of plants^20^. Peroxidase is involved in physiological and biochemical processes in plants.It regulates the development process of higher plants, contains iron porphyrin cofactors, and closely participates in cell development. The occurrence and development of adventitious roots in plants are related to the action of catalase^21^.

Superoxide dismutase (SOD) is a kind of metal enzyme widely existing in microorganisms, animal and plant cells. In plant cells, it can protect against reactive oxygen species and other external interference in a high potassium environment^22^. Acyl coenzyme A synthetase is involved in fatty acid metabolism. It can activate free fatty acids to Acyl coenzyme a thiolipids. Sulfur lipids can participate in signaling, transcriptional regulation, transmembrane transport and other metabolic pathways, and they are the substrates of β-oxidation^23^. Our results that these enzymes are more abundant under high potassium conditions indicates that the growth and development of roots are promoted at high potassium levels.

### Differentially abundant proteins related to genetic information processing

Histones are related to chromosome replication in the cell cycle or are useful in gene expression regulation^24^. Our results showed that compared with the control treatment, differentially up-regulated proteins at high potassium that play a role in gene expression regulation are histones, ribosomal proteins, RNA helicase, etc (Fig. 2). These enzymes are up-regulated and are involved in genetic information processing of tobacco roots in a high potassium environment. The up regulation of ribosomal protein is very obvious (21.21 times), which means that in the high potassium treatment, the protein synthesis related pathway is strengthened. This is more conducive to the growth of tobacco roots. The up regulation of RNA helicase will promote protein synthesis and accelerate the movement of proteins in the process of root development.

### Differentially abundant proteins related to cell processes and environmental information processing

Sucrose synthetase is an important enzyme of sucrose metabolism, which plays a vital role in plant growth. It can control sucrose metabolism and sucrose accumulation, provide energy for plant growth and development, and regulate cell metabolism processes^25^. Our results showed that the inhibitory factors of sucrose synthetase and K^+^ transport growth defect (up-regulated by 2.04 times) were up-regulated, and the expression of apoptosis ATPase was down regulated under high potassium treatment. This may be due to the high affinity of tobacco for potassium, The up-regulation of K^+^ transporter or K^+^ channel related protein, and the up-regulation of K^+^ transport growth defect inhibitory factor indicate that the root system needs better growth and development in an environment with high K^+^ concentration. The down regulation of apoptotic ATPase may be due to the energy demand of tobacco's faster growth and development.

### Other differentially abundant proteins

Chitinase is an important plant defense factor; it plays an important role in plant growth and development, stress resistance and defense response^26^. ABC transporter has a transmembrane domain, which can be used as a membrane integrin to transport peptides, sugars, lipids, heavy metal chelates and other organic compounds^27^. Our results showed that compared with the control treatment, the other differentially abundant proteins in the high potassium treatment were chitinase, high glucose regulatory enzyme, conserved protein, ABC transporter and GTP enzyme. All of these enzymes were up-regulated. This indicates that in the high potassium treatment, due to the enhancement of metabolic pathways, the high glucose regulatory enzyme was up-regulated, as well as the up-regulated conserved protein (9.79 times). GTP can promote the development and growth of tobacco roots, and can provide energy for the material metabolism of tobacco growth.

## Conclusion

In summary, our findings suggest that in the high potassium environment the root differentially abundant proteins of flue-cured tobacco seedlings are mainly located in the cell component, the organelle component and the membrane organelle. The proteins we identified have molecular functions such as hydrolase activity, ion binding, small molecule binding, heterocyclic and organic ring compound binding, and participate in biological processes such as monomer metabolism, cell metabolism, organic acid metabolism, basic metabolism, monomer cell process and nitrogen component metabolism.

The proteins in tobacco plants under high potassium treatment that are involved in transformation and translation are easier to express differentially and mainly participated in material metabolism and other related pathways. Meanwhile they also play a key role in promoting the growth and development of the tobacco root system.

## Supplementary Information

Supplementary Information
