# Supplementary material for: Differential proteomics of tobacco seedling roots at high and low potassium concentrations
Source: Sci Rep. 2021 Apr 28;11:9194. doi: 10.1038/s41598-021-88689-4 (PMC8080629; doi:10.1038/s41598-021-88689-4)
Supplement: Supplementary file 1 — Supplementary Information [file 41598_2021_88689_MOESM1_ESM.docx]

**Supplementary materials**


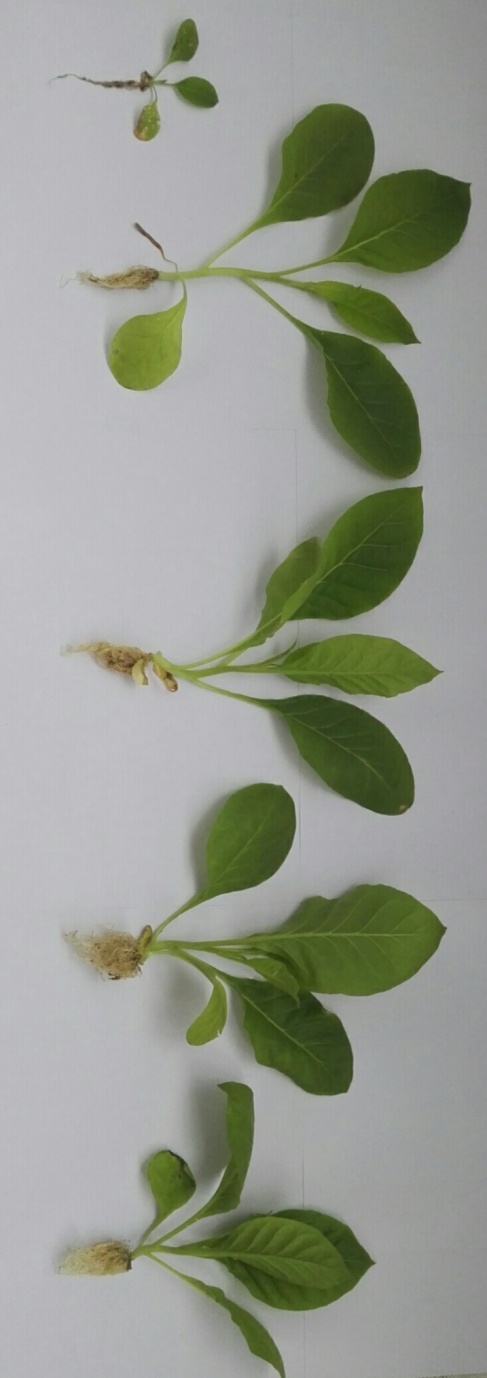
**Explain one:** Because the submitted paper used normal potassium concentration and 720 high potassium concentration, the original pictures (figure 1)were processed picture after processing(figure 2).

Note :The order is low potassium treatment([K^+^]=0 mg · L^-1^)，normal potassium treatment([K^+^]=240 mg·L^-1^) , high potassium treatment 1([K^+^]=360mg·L^-1^),high potassium treatment 2([K^+^]=480mg·L^-1^) ,high potassium treatment 3([K^+^]=720 mg · L^-1^) from left to right.

Figure 1 Picture before processing （original picture）


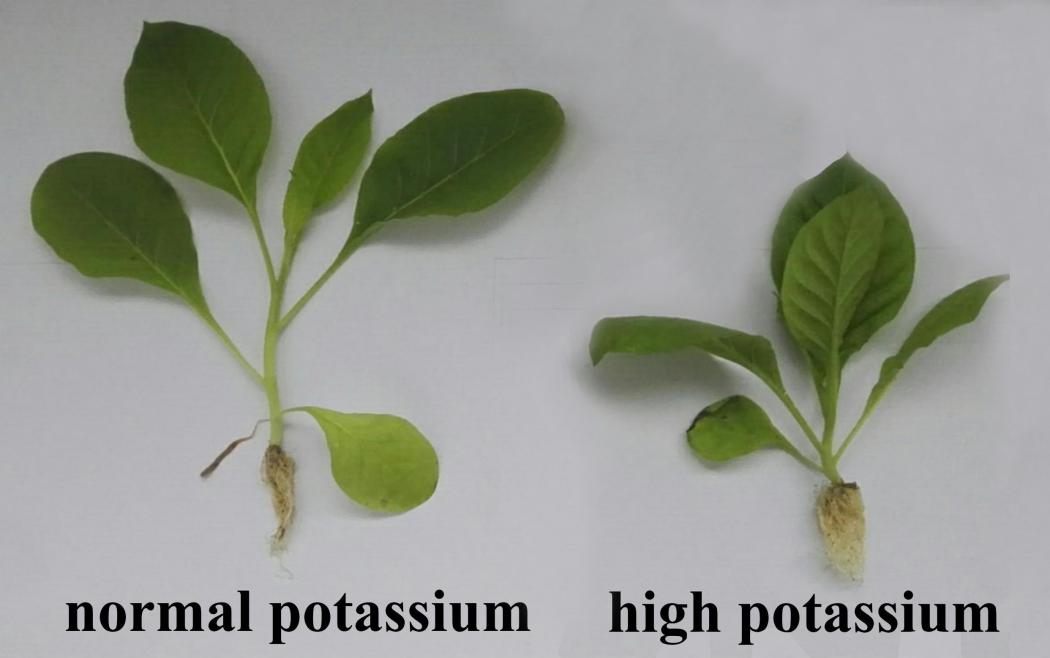


Figure 2 Picture after processing （submitted picture）

**Explain two:** Protein electrophoretic map of the original picture is figure 3(Picture before processing), the paper submitted picture is figure 3 (Picture after processing).


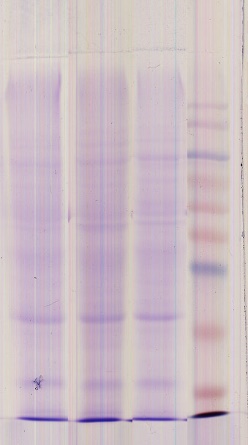


Figure 3 Picture before processing （original picture）

Note :The order is low potassium treatment([K^+^]=0 mg · L^-1^)，normal potassium treatment([K^+^]=240 mg·L^-1^) , high potassium treatment ([K^+^]=720 mg · L^-1^) from left to right.


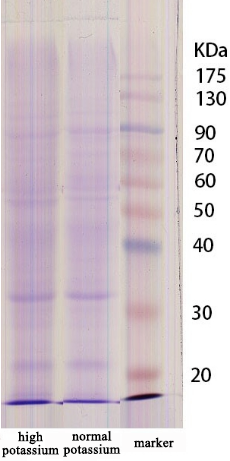


Figure4 Picture after processing （submitted picture）
